# Transcranial Sonographic Characteristics of Substantia Nigra in End-Stage Renal Disease Patients with Restless Legs Syndrome: A Diagnostic Marker Study

**DOI:** 10.3390/diagnostics16010041

**Published:** 2025-12-22

**Authors:** Caishan Wang, Zhoubing Zhan, Changwei Ding, Yingchun Zhang, Weifeng Luo

**Affiliations:** 1 Department of Ultrasound, The Second Affiliated Hospital of Soochow University, Suzhou 215004, China; wcs9422@suda.edu.cn (C.W.); dd15755386072@163.com (C.D.); richer777@126.com (Y.Z.); 2 Department of Nephrology, The Second Affiliated Hospital of Soochow University, Suzhou 215004, China; zhanzhoubing@foxmail.com; 3 Department of Neurology and Clinical Research Centre of Neurological Disease, The Second Affiliated Hospital of Soochow University, Suzhou 215004, China

**Keywords:** end-stage renal disease, transcranial sonography, restless legs syndrome, substantia nigra, diagnostic marker

## Abstract

**Objective**: Restless legs syndrome (RLS) is a highly prevalent neurological complication in end-stage renal disease (ESRD) patients. This study aimed to explore the transcranial sonography (TCS) characteristics of the substantia nigra (SN) and brainstem raphe (BR) in ESRD patients with and without RLS and to evaluate the diagnostic value of SN echogenicity for ESRD-related RLS. **Methods**: A total of 65 ESRD patients (45 with RLS [ESRD + RLS] and 20 without RLS [ESRD − RLS]) from the dialysis center and 30 age- and gender-matched healthy controls (NC) from the health management center were enrolled between January 2017 and December 2022. All participants underwent TCS to measure the bilateral SN echogenic area, and the total SN echogenic area (SNsA) was calculated. BR echogenicity was assessed using a semiquantitative scale. Receiver operating characteristic (ROC) curves were plotted to determine the optimal SNsA cutoff for diagnosing ESRD + RLS. **Results**: The SNsA in the ESRD + RLS group [0.15 (0.13–0.22) cm^2^] was significantly smaller than that in the ESRD − RLS group [0.27 (0.23–0.31) cm^2^] and the NC group [0.27 (0.22–0.30) cm^2^] (both *p* < 0.001). ROC curve analysis showed that SNsA had the highest diagnostic efficacy for ESRD + RLS, with an area under the curve (AUROC) of 0.823 (95% confidence interval [CI]: 0.722–0.924). At a cutoff of 0.22 cm^2^, SNsA yielded a sensitivity of 85.0%, specificity of 73.3%, accuracy of 76.92%, positive predictive value (PPV) of 58.6%, and negative predictive value (NPV) of 91.7%. The prevalence of BR hypoechogenicity was significantly higher in ESRD + RLS (33.33%) and ESRD − RLS (35.00%) groups than in the NC group (10.00%) (both *p* < 0.05), but no difference was observed between the two ESRD subgroups (*p* > 0.05). No significant differences in third ventricle (TV) width or bilateral middle cerebral artery peak systolic velocity (MCA-PSV) were found among the three groups (all *p* > 0.05). **Conclusions**: ESRD + RLS patients exhibit significant SN hypoechogenicity compared with ESRD − RLS patients and healthy controls. SNsA with a cutoff of 0.22 cm^2^ serves as a reliable imaging biomarker for diagnosing ESRD + RLS, and TCS is a valuable noninvasive tool to assist clinical decision-making in this population.

## 1. Introduction

Restless legs syndrome (RLS) is a sensorimotor neurological disorder characterized by an irresistible urge to move the lower limbs, often accompanied by uncomfortable paresthesias [1]. Clinically, RLS is classified into primary (idiopathic) and secondary subtypes, with ESRD-related RLS (ESRD + RLS) being one of the most common secondary forms. Clinically, RLS is classified into primary (idiopathic) and secondary subtypes, with ESRD-related RLS (ESRD + RLS) being one of the most common secondary forms. Previous studies have reported that the prevalence of RLS in ESRD patients varies widely across regions, ranging from 15.8% (Iran) to 52.6% (Brazil), with common rates of 20–26% in many cohorts (e.g., 20.44% in mainland China, 22% in Japan, 25.3% in Taiwan, 26.6% in Greece), which is substantially higher than the 7.2% prevalence in the general Chinese population [2].

ESRD + RLS imposes a significant burden on patients, as it coexists with renal dysfunction and is associated with mental fatigue, depression, poor sleep quality, physical exhaustion, and muscle atrophy—all of which severely reduce quality of life [3,4,5]. However, the diagnosis of ESRD + RLS currently relies solely on clinical manifestations and response to dopamine agonists. There are no reliable physical signs, imaging biomarkers, or laboratory indicators to reflect the pathological changes in ESRD + RLS, leading to high rates of underdiagnosis and inadequate treatment.

Transcranial sonography (TCS) is a noninvasive, cost-effective imaging technique that can clearly visualize echogenicity changes in the midbrain and basal ganglia [6]. It has been increasingly used in the evaluation of neurodegenerative disorders, particularly Parkinson’s disease (PD), where SN hyperechogenicity is a classic diagnostic feature [7,8,9,10]. In contrast, recent studies on idiopathic RLS (iRLS) have demonstrated significant SN hypoechogenicity compared with both PD patients and normal controls, suggesting that SN hypoechogenicity may be a key morphological marker for iRLS [11,12]. The mechanism underlying SN echogenicity changes is closely linked to tissue iron content: hyperechogenicity reflects increased iron accumulation (e.g., in PD), while hypoechogenicity indicates iron deficiency [13,14]—a well-established pathophysiological factor in RLS.

To our knowledge, no previous studies have investigated the echogenicity characteristics of SN in ESRD + RLS. Given the high prevalence of ESRD + RLS and the lack of reliable diagnostic markers, this study aimed to: (1) compare TCS findings (including SN echogenicity and BR changes) among ESRD + RLS, ESRD − RLS, and healthy control groups; (2) establish a diagnostic cutoff value for SN echogenicity in ESRD + RLS; and (3) explore the potential of TCS as a noninvasive diagnostic tool for ESRD + RLS.

## 2. Materials and Methods

### 2.1. Study Participants

Consecutive ESRD patients were enrolled from the Dialysis Center of the Second Affiliated Hospital of Soochow University between January 2017 and December 2022. ESRD was diagnosed according to the Chronic Kidney Disease Epidemiology Collaboration (CKD-EPI) formula [15], which estimates glomerular filtration rate. Patients with an estimated glomerular filtration rate of <15 mL/min/1.73 m^2^ and who had been undergoing regular peritoneal dialysis or hemodialysis for ≥3 months were included.

A total of 78 ESRD patients were initially screened and divided into two subgroups based on RLS diagnosis: ESRD + RLS (*n* = 52) and ESRD − RLS (*n* = 26).

Inclusion criteria for ESRD patients: Aged ≥18 years; For ESRD + RLS: Diagnosis of RLS based on the 2014 IRLSSG criteria; For ESRD − RLS: No history of RLS symptoms and no RLS diagnosis by a neurologist; Ability to understand and complete study-related assessments.

Exclusion criteria: Primary RLS (diagnosed before ESRD onset); Neurodegenerative diseases (e.g., PD) or movement disorders; Poor temporal bone window sound transmission (preventing clear TCS visualization of the midbrain); Severe cardiopulmonary, hepatic, or hematological diseases.

Thirty-five age- and gender-matched healthy controls were recruited from the hospital’s Health Management Center. Among them, 5 were excluded due to inadequate TCS imaging quality, leaving 30 controls (17 males, 18 females; mean age: 63.2 ± 5.3 years, range: 54–78 years). All controls had no history of RLS, kidney disease, neurodegenerative disease, or motor neuron disease.

This study was approved by the Ethics Committee of the Second Affiliated Hospital of Soochow University (approval number: JD-LK-2018-094-01) and conducted in accordance with the Declaration of Helsinki. All participants or their legal representatives provided written informed consent.

### 2.2. RLS Diagnostic Criteria

RLS was diagnosed strictly based on the 2014 IRLSSG criteria [16], which require the simultaneous presence of the following five items: An irresistible urge to move the lower limbs, often accompanied by lower limb discomfort; The urge to move or discomfort worsens at rest (e.g., sitting, lying down); The discomfort is partially or completely relieved by activity (e.g., walking, stretching); The urge to move or discomfort is exacerbated or occurs exclusively in the evening/night; Exclusion of other conditions causing lower limb discomfort (e.g., muscle pain, venous obstruction, edema, arthritis, habitual foot-tapping).

### 2.3. TCS Examination Protocol

TCS images were acquired using an Acuson Sequoia 512 Ultrasound System (Siemens Medical Solutions Inc., Mountain View, CA, USA) equipped with a 2.5 MHz 4V1C transducer. Two operators with more than 5 years of TCS experience performed the examinations, and both were blinded to the participants’ clinical information (e.g., RLS diagnosis, ESRD subtype). The protocol followed guidelines from the Ninth Meeting of the European Society of Neurosonology and Cerebral Hemodynamics, supplemented by the operators’ clinical experience [17].

Ultrasound parameters: Frequency: 2.5 MHz; Depth: 14–16 cm; Dynamic range: 45–55 dB; Image contrast, brightness, and time gain compensation were adjusted to ensure optimal visualization of midbrain structures. In the standard midbrain plane, the midbrain appears as a low-echogenicity “butterfly-shaped” structure, surrounded by the markedly hyperechogenic basal cistern (BC). The BR is visualized as a linear, high-echogenicity midline structure, while the SN is located bilaterally in the ventrolateral midbrain.

### 2.4. TCS Image Analysis:

SN echogenic area measurement: SN echogenic area refers to the midbrain substantia nigra region that exhibits significantly higher echogenicity than the surrounding brain tissue. The echogenic area of each SN was manually traced in the midbrain plane (ipsilateral to the probe) by operators using the built-in measurement software of the ultrasound system. Upon completion of tracing, the ultrasound device or image analysis software automatically calculates the area of the substance echogenicity region. The total SN echogenic area (SNsA) was calculated as the sum of bilateral SN areas.

BR semiquantitative assessment: The BR was evaluated on the side with the most precise visualization using a 2-point scale: Grade 1 (normal)—Continuous midline echogenicity; Grade 0 (hypoechogenic)—Reduced, interrupted, or absent echogenicity.

Additional measurements: Included Bilateral MCA-PSV and TV width, which were assessed to evaluate cerebral hemodynamics and midbrain atrophy, respectively.

### 2.5. Statistical Method

All analyses were performed using R version 4.0 (https://www.r-project.org/, accessed on 10 October 2025). The Shapiro–Wilk test was used to assess the normality of continuous data. Normally distributed data were expressed as mean ± standard deviation (SD), and non-normally distributed data as median (interquartile range [IQR]). Categorical data were presented as counts (percentages). Between two groups: Independent samples *t*-test (normal distribution) or Mann–Whitney U test (non-normal distribution); Among three groups: One-way ANOVA (normal) or Kruskal–Wallis test (non-normal) for continuous data; chi-squared test or Fisher’s exact test for categorical data—post hoc comparisons: Bonferroni correction for multiple pairwise comparisons. The intraclass correlation coefficient (ICC) was used to evaluate the interobserver variability in the SN echogenic areas on both sides, as measured by the two sonographers. The ICC was rated as poor (0.00–0.20), fair (0.20–0.40), good (0.40–0.75), or excellent (0.75–1.00).

Spearman’s correlation analysis was used to explore associations between SNsA and clinical parameters (age, disease duration) in ESRD + RLS. ROC curve analysis was performed to evaluate the diagnostic value of SNsA for ESRD + RLS, with the optimal cutoff determined by the Youden index. The significance level was set at α = 0.05.

## 3. Results

### 3.1. Participant Disposition and Baseline Characteristics

Of the 113 initially recruited subjects, 18 (15.9%) were excluded due to poor temporal bone window sound transmission and inability to identify SN echogenicity: 13 (25.0%) from the ESRD group and 5 (14.3%) from the control group. The final analysis included 65 ESRD patients (45 ESRD + RLS, 20 ESRD − RLS) and 30 healthy controls. The ICCs for interobserver variability in measuring the RSN and LSN were 0.85 (95% confidence interval: 0.56–0.95) and 0.83 (95% confidence interval: 0.51–0.92), respectively.

Table 1 presents the baseline clinical characteristics and TCS findings of the three groups. There were no significant differences in age (*p* = 0.975) or gender distribution (*p* = 0.726) among the groups. However, the ESRD + RLS group had a significantly longer dialysis duration than the ESRD − RLS group (67.93 ± 16.19 vs. 45.25 ± 18.49 months, *p* < 0.001).

### 3.2. TCS Findings

#### 3.2.1. SN Echogenicity

**Figure 1:**
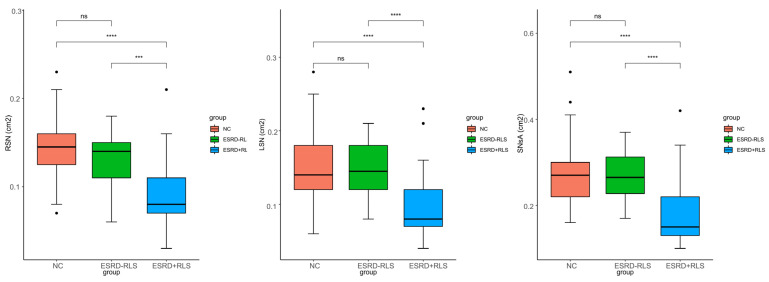
Comparison of the RSN, LSN, and SNsA in ESRD + RLS patients, ESRD − RLS patients, and NC. *: *p* < 0.05; **: *p* < 0.01; ***: *p* < 0.001; ****: *p* < 0.0001. ESRD + RLS: ESRD patients with restless leg syndrome; ESRD − RLS: ESRD patients without restless leg syndrome; NC: normal control; RSN: right substantia nigra; LSN: left substantia nigra, SNsA: the sum value of SN areas of echogenicity of both sides.

Significant differences in bilateral SN area (RSN, LSN) and SNsA were observed among the three groups (all *p* < 0.001; Figure 1). Post hoc analysis showed that RSN, LSN, and SNsA were significantly smaller in the ESRD + RLS group than in the ESRD − RLS and normal control groups (all *p* < 0.001), but no differences were found between the ESRD − RLS and control groups (all *p* > 0.05).

**Figure 2:**
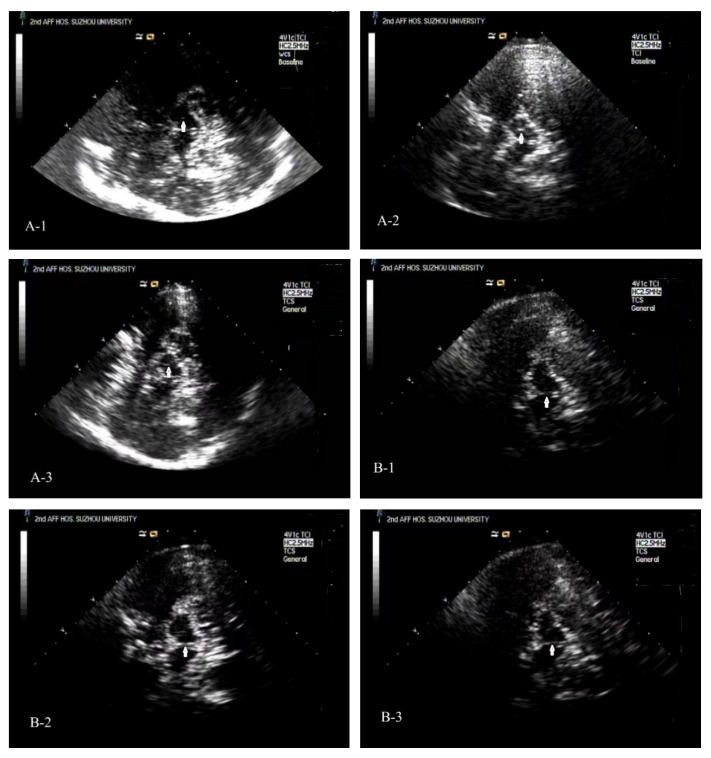
Typical TCS images of the midbrain plane of ESRD + RLS, ESRD − RLS patient, and NC. (**A-1**): A TCS image of the midbrain plane of an ESRD + RLS shows that the area of ipsilateral SN echogenicity was 0.07 cm^2^. (**A-2**): A TCS image of the midbrain plane of a normal control reveals that the area of ipsilateral SN echogenicity was 0.14 cm^2^. (**A-3**): A TCS image of the midbrain plane of an ESRD − RLS reveals that the area of ipsilateral SN echogenicity was 0.12 cm^2^. (**B-1**): A TCS image of the midbrain plane of an ESRD + RLS patient shows interrupted brainstem raphe echogenicity; (**B-2**): A TCS image of the brainstem raphe of a normal control exhibits continuous echogenicity. (**B-3**): A TCS image of the brainstem raphe of an ESRD − RLS exhibits interrupted brainstem raphe echogenicity.

**Figure 3:**
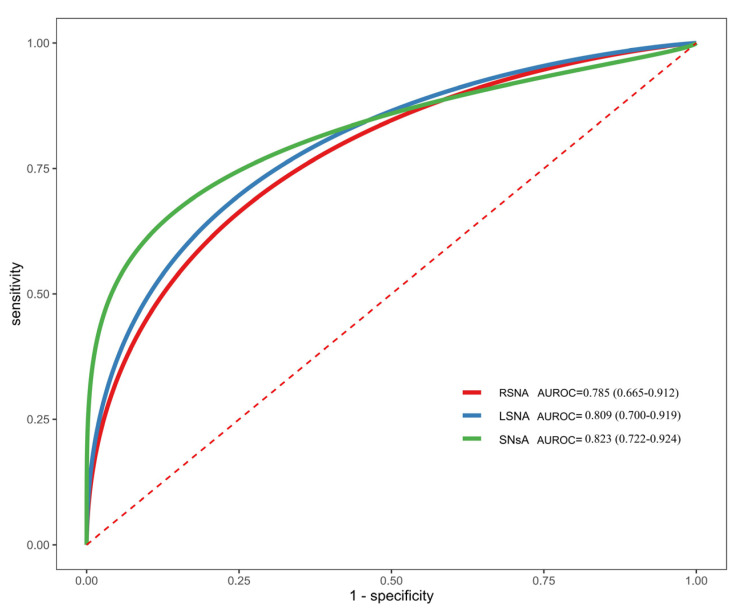
ROC curves of RSN, LSN, and SNsA in distinguishing between ESRD + RLS and ESRD − RLS. ROC: receiver operating characteristic curve. RSN: right substantia nigra; LSN: left substantia nigra; SNsA: the sum value of SN areas of echogenicity of both sides.

Typical TCS images of the midbrain plane of ESRD + RLS, ESRD − RLS patient, and NC [Figure 2(A-1–A-3)].

#### 3.2.2. BR Echogenicity

The prevalence of BR hypoechogenicity (Grade 0) was 33.33% in ESRD + RLS, 35.00% in ESRD − RLS, and 10.00% in controls. Both ESRD subgroups had significantly higher rates of BR hypoechogenicity than controls (ESRD + RLS vs. controls: *p* = 0.048; ESRD − RLS vs. controls: *p* = 0.037), but no difference was noted between ESRD + RLS and ESRD − RLS (*p* > 0.05). Typical TCS images could see Figure 2(B-1–B-3).

#### 3.2.3. TV Width and MCA-PSV

No significant differences were observed in TV width, R-MCA-PSV, or L-MCA-PSV among the three groups (all *p* > 0.05).

### 3.3. Diagnostic Value of SNsA for ESRD + RLS

ROC curve analysis was performed to evaluate the diagnostic efficacy of RSN, LSN, and SNsA for distinguishing ESRD + RLS from ESRD − RLS (Figure 3). SNsA exhibited the highest diagnostic value, with an AUROC of 0.823 (95% CI: 0.722–0.924). At a cutoff of 0.22 cm^2^, SNsA had a diagnostic accuracy of 76.92%, sensitivity of 85.0%, specificity of 73.3%, PPV of 58.6%, and NPV of 91.7% (Table 2). The confusion matrix for this cutoff is shown in Table 3.

### 3.4. Correlation Between SNsA and Clinical Parameters

**Figure 4:**
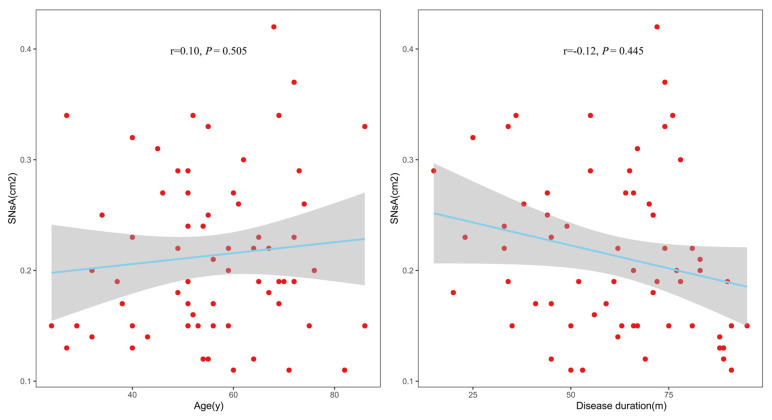
Correlations of SNsA with age and disease duration in ESRD + RLS. SNsA: the sum value of the SN areas of echogenicity of both sides.

In the ESRD + RLS group, Spearman’s correlation analysis showed no significant associations between SNsA and age (r = 0.10, *p* = 0.505) or ESRD duration (r = −0.12, *p* = 0.445); see Figure 4.

## 4. Discussion

This study is the first to systematically investigate TCS characteristics in ESRD + RLS patients, demonstrating three key findings: (1) ESRD + RLS patients have significantly smaller SN echogenic areas (SNsA) than ESRD − RLS patients and healthy controls; (2) SNsA with a cutoff of 0.22 cm^2^ has good diagnostic value for ESRD + RLS (AUROC = 0.823); (3) Both ESRD subgroups have higher rates of BR hypoechogenicity than controls, but this finding is not specific to RLS. These results provide novel insights into the pathophysiology of ESRD + RLS and support TCS as a noninvasive diagnostic tool for this population.

In this study, the ESRD + RLS group (*n* = 45) had more than twice as many patients as the ESRD − RLS group (*n* = 20), leading to an imbalance in group sizes and compromising the robustness of the diagnostic cutoff. Group imbalance can cause classifiers to overemphasize the majority class in imbalanced datasets, potentially leading to underrepresentation of minority instances or misclassifying them as noise. This study primarily aims to diagnose the ESRD + RLS group (the majority class) from all ESRD patients, which has relatively limited influence. In addition, in this study, we not only estimated the ROC curve and AUC value but also comprehensively assessed the diagnostic cutoff performance using metrics such as precision, recall, and F1 score.

### 4.1. SN Hypoechogenicity: A Biomarker for ESRD + RLS

SN echogenicity is closely associated with tissue iron content [13,14]. Since ultrasound waves usually reflect at tissue interfaces or from larger molecules, it can be hypothesized that only iron bound in a specific manner or to specific proteins reflects. Previous studies have shown that SN hyperechogenicity in PD is due to increased iron accumulation, while SN hypoechogenicity in iRLS reflects iron deficiency [11,18]. Our study found that ESRD + RLS patients have a significantly smaller SNsA than ESRD − RLS patients and controls, suggesting that ESRD + RLS may share a similar pathophysiological mechanism with iRLS, specifically cerebral iron deficiency.

The link between ESRD and SN iron deficiency may be multifactorial. However, this study did not investigate changes in iron metabolism data, such as ferritin and total serum iron, in ESRD patients. Kalantar-Zadeh K et al. have demonstrated that ESRD patients often suffer from chronic inflammation, which impairs iron absorption and increases iron sequestration in macrophages [19]. Additionally, hemodialysis can lead to iron loss through blood cell damage and adsorption by the dialysis membrane [20]. Iron is essential for dopamine synthesis and neuronal function in the SN; deficiency may disrupt dopamine signaling, contributing to RLS symptoms. This hypothesis is supported by previous studies showing that iron supplementation improves RLS symptoms in ESRD patients [21]. Based on the above analysis, we can infer that SN hypoechogenicity, consistent with decreased regional iron content, may indicate more pronounced brain iron deficiency in ESRD + RLS than in ESRD − RLS.

Notably, SNsA was not correlated with age or ESRD duration in the ESRD + RLS group, indicating that SN hypoechogenicity is not a progressive change associated with disease chronicity. Instead, it may serve as a preclinical biomarker for ESRD + RLS, facilitating early diagnosis before the onset of severe symptoms.

### 4.2. Diagnostic Value of SNsA

The ROC curve analysis showed that SNsA has good diagnostic efficacy for ESRD + RLS, with an AUROC of 0.823. At a cutoff of 0.22 cm^2^, the sensitivity (85.0%) and NPV (91.7%) are particularly high, indicating that SNsA < 0.22 cm^2^ strongly suggests ESRD + RLS. This cutoff is consistent with previous studies on iRLS, where SNsA cutoffs for distinguishing iRLS from controls ranged from 0.16 to 0.23 cm^2^. The consistency in cutoffs further supports the hypothesis that ESRD + RLS and iRLS share a common pathophysiological basis.

The high NPV of SNsA is clinically valuable, as it can help rule out ESRD + RLS in patients with SNsA ≥ 0.22 cm^2^, thereby reducing unnecessary treatment. The moderate specificity (73.3%) and PPV (58.6%) may be due to the small sample size and potential overlap in SN echogenicity between ESRD + RLS and ESRD − RLS patients. Future studies with larger cohorts are needed to validate the cutoff and improve diagnostic accuracy.

### 4.3. BR Hypoechogenicity in ESRD Patients

Godau et al. reported that 75% of iRLS patients have BR hypoechogenicity, suggesting this as a complementary marker for iRLS [22]. However, our study found similar BR hypoechogenicity rates in the ESRD + RLS (33.33%) and ESRD − RLS (35.00%) groups, both of which were significantly higher than in the controls (10.00%). This discrepancy may be explained by the high prevalence of depression in ESRD patients—previous studies have shown that BR hypoechogenicity is a TCS marker for depression, as reduced BR continuity reflects serotonergic dysfunction [23]. Depression is equally common in ESRD + RLS and ESRD − RLS patients, which may explain the similar BR hypoechogenicity rates. Thus, BR hypoechogenicity in ESRD patients may be associated with depression rather than RLS itself, limiting its diagnostic value for ESRD + RLS.

The 5-hydroxytryptamine (5-HT) nerve fibers originating from the BR primarily project to the anterior limbic system and the cerebral cortex. In patients with depression, the pathological basis of the BR hypoechogenicity in the TCS image may be attributed to the degeneration and necrosis of the dorsal nucleus of the BR, which has been confirmed by MRI [24,25].

### 4.4. Limitations

This study has several limitations. First, it was a single-center study with a relatively small sample size (*n* = 95), which may limit the generalizability of our findings. Multi-center studies with larger cohorts are needed to validate the SNsA cutoff and confirm the results. Second, TCS is highly dependent on temporal bone window quality; elderly females were more likely to be excluded due to postmenopausal osteoporosis [26], potentially introducing a selection bias. Third, we did not assess participants’ iron metabolism parameters (e.g., serum ferritin, transferrin saturation) or depressive symptoms, which prevented further analysis of the associations between SN echogenicity, iron status, and depression. Fourth, the follow-up period was not reported; future studies should evaluate the long-term prognostic value of SNsA for ESRD + RLS. Lastly, since we did not conduct subgroup analyses by dialysis modality (hemodialysis vs. peritoneal dialysis), whether dialysis modality influences TCS findings remains to be investigated.

## 5. Conclusions

ESRD + RLS patients exhibit significant SN hypoechogenicity compared with ESRD − RLS patients and healthy controls. SNsA with a cutoff of 0.22 cm^2^ serves as a reliable imaging biomarker for diagnosing ESRD + RLS, and TCS is a valuable noninvasive tool to assist in clinical decision-making. The findings suggest that ESRD + RLS may share a similar pathophysiological mechanism with iRLS, specifically cerebral iron deficiency. Future studies should investigate the association between SN echogenicity and iron metabolism parameters and validate the diagnostic value of SNsA in multi-center cohorts.

## Figures and Tables

**Table 1:** (**A**) Clinical characteristics and TCS findings of ESRD + RLS, ESRD − RLS, and normal controls. (**B**) Post hoc values using Bonferroni correction for Clinical characteristics and TCS findings among ESRD + RLS, ESRD − RLS, and normal controls.

(A)				
	ESRD + RLS (N = 45)	ESRD − RLS (N = 20)	NC (N = 30)	*p*
Age (y)	55.78 ± 14.95	55.55 ± 14.56	55.00 ± 14.21	0.975 ^a^
Sex				0.726 ^b^
F	17 (37.78%)	6 (30.00%)	9 (30.00%)	
M	28 (62.22%)	14 (70.00%)	21 (70.00%)	
Disease duration (month)	67.93 ± 16.19	45.25 ± 18.49		<0.001 ^c^
TV (cm)	0.510 (0.40–0.65)	0.610 (0.53–0.70)	0.55 (0.36–0.69)	0.091 ^d^
R-MCA-PSV (cm/s)	92.00(76.00–109.00)	90.500 (72.75–121.75)	93.00(81.50–107.75)	0.942 ^d^
L-MCA-PSV (cm/s)	96.00 (80.00–112.00)	91.50 (84.00–125.750)	87.50(79.25–110.00)	0.681 ^d^
BR				0.049 ^b^
1	30 (66.67%)	13 (65.00%)	27 (90.00%)	
0	15 (33.33%)	7 (35.00%)	3 (10.00%)	
RSN (cm^2^)	0.08 (0.07–0.11)	0.14 (0.11–0.15)	0.15 (0.13–0.16)	<0.001 ^d^
LSN (cm^2^)	0.08 (0.07–0.12)	0.15 (0.120–0.180)	0.14 (0.12–0.18)	<0.001 ^d^
SNsA (cm^2^)	0.15 (0.13–0.22)	0.27 (0.23–0.312)	0.27 (0.22–0.30)	<0.001 ^d^
(**B**)				
	***p* (NC vs. ESRD − RLS)**	***p* (NC vs. ESRD + RLS)**	***p* (ESRD − RLS vs. ESRD + RLS)**	
Age (y)	0.991 ^e^	0.972 ^e^	0.998 ^e^	
sex	1.000 ^e^	1.000 ^e^	1.000 ^e^	
TV (cm)	0.127 ^e^	0.935 ^e^	0.092 ^e^	
R-MCA-PSV (cm/s)	0.960 ^e^	0.960 ^e^	0.960 ^e^	
L-MCA-PSV (cm/s)	0.678 ^e^	0.678 ^e^	0.926 ^e^	
BR	0.101 ^e^	0.101 ^e^	1.000 ^e^	
RSN (cm^2^)	0.117 ^e^	<0.001 ^e^	<0.001 ^e^	
LSN (cm^2^)	0.674 ^e^	<0.001 ^e^	<0.001 ^e^	
SNsA (cm^2^)	0.827 ^e^	<0.001 ^e^	<0.001 ^e^	

Data are given as n (%), mean ± SD, and median (Q1, Q3); ^a^ ANOVA test; ^b^ chi-squared test; ^c^ Student’s *t*-test; ^d^: Kruskal–Wallis test; ^e^: Post hoc tests using Bonferroni correction. ESRD + RLS: ESRD patients with restless leg syndrome; ESRD − RLS: ESRD patients without restless leg syndrome; NC: normal control; TV: third ventricle; R-MCA-PSV: right middle cerebral artery peak flow velocity; L-MCA-PSV: left middle cerebral artery peak flow velocity; BR: raphe nuclei, BR: midbrain raphe; RSN: right substantia nigra; LSN: left substantia nigra, SNsA: the sum value of SN areas of echogenicity of both sides.

**Table 2:** Analysis of diagnostic efficacy for hyperechogenicity area of LSN, RSN, and SNsA sizes.

	AUROC	SE	SP	PPV	NPV	Cutoff Value(cm^2^)
RSN	0.785 (0.665–0.912)	0.700	0.822	0.636	0.860	0.12
LSN	0.809 (0.700–0.919)	0.650	0.844	0.650	0.844	0.14
SNsA	0.823(0.7220–0.924)	0.850	0.733	0.586	0.917	0.22

AUROC, area under the receiver operating characteristic curve; ACC: Accuracy; SE: Sensitivity; SP: Specificity; PPV: Positive predictive value; NPV: Negative predictive value. RSN: right substantia nigra; LSN: left substantia nigra; SNsA: the sum value of SN areas of echogenicity of both sides.

**Table 3:** The confusion matrix for predicting ESRD + RLS with the SNsA threshold set at 0.22 cm^2^.

Groups	ESRD − RLS (*n* = 20)	ESRD + RLS (*n* = 45)
SNsA < 0.22(cm^2^)	3	33
SNsA ≥ 0.22 (cm^2^)	17	12

ESRD + RLS: ESRD patients with restless leg syndrome; ESRD − RLS: ESRD patients without restless leg syndrome. SNsA: the sum value of SN areas of echogenicity of both sides.

## Data Availability

The data generated and analyzed during the current study contain sensitive personal health information of participants, including clinical characteristics, dialysis-related data, and transcranial sonography (TCS) imaging data linked to individual patient identities. In accordance with the MDPI Research Data Policies (https://www.mdpi.com/ethics, accessed on 16 November 2025), and to strictly protect patient privacy, this data cannot be publicly shared—consistent with the ethical approval requirements of the Ethics Committee of the Second Affiliated Hospital of Soochow University (approval number: JD-LK-2018-094-01) and relevant national medical data privacy regulations.

## References

[1] Anders B., Zainab A., Jonas L., Martin U., Fredrik L., Amir P. (2023). Worldwide estimation of restless legs syndrome: A systematic review and meta-analysis of prevalence in the general adult population. J. Sleep Res..

[2] Lin X.-W., Zhang J.-F., Qiu M.-Y., Ni L.-Y., Yu H.-L., Kuo S.-H., Ondo W.G., Yu Q. (2019). Restless legs syndrome in end stage renal disease patients undergoing hemodialysis. BMC Neurol..

[3] Mahjabeen Y., Furqan A.J., Sadia Y., Hassan A.S., Hamza M., Sadiq N. (2021). Association of quality of life, anxiety, and depression with restless leg syndrome in the hemodialysis patients. BMC Res. Notes.

[4] Anupama A.V., Mehta A., Javali M., Eswarappa M., Rangaiah P., Acharya P. (2025). Prevalence, risk factors, and psychosocial impact of restless legs syndrome in end-stage renal disease patients undergoing hemodialysis—A cross-sectional study. Ann. Indian Acad. Neurol..

[5] Liu Y., Du Q., Jiang Y. (2024). Prevalence of restless legs syndrome in maintenance hemodialysis patients: A systematic review and meta-analysis. Sleep Med..

[6] Stiehm M., Nilsson C., Skogar Ö., Walter U. (2025). The diagnostic value of transcranial sonography in Swedish parkinsonism patients: A retrospective cohort study with long-term follow-up. Clin. Park. Relat. Disord..

[7] Kelson J.A., Edson B., José L.P., Andre C.F., Marcelo L.O., Orlando G.P., Ricardo C.N., Fernando M.P., Vanderci B., Ilza R.B. (2022). Combined assessment by transcranial sonography and Sniffin’ Sticks test has a similar diagnostic accuracy compared to brain SPECT for Parkinson’s disease diagnosis. Clin. Neurol. Neurosurg..

[8] Zhang Y.-Y., Jiang X.-H., Zhu P.-P., Zhuo W.-Y., Liu L.-B. (2024). Advancements in understanding substantia nigra hyperechogenicity via transcranial sonography in Parkinson’s disease and its clinical implications. Front. Neurol..

[9] Kammineni A., MS D., Borgohain R., Jabeen S.A., Kandadai R.M., Jyotsna Y., Jayalakshmi B., Srilatha B.N. (2025). To determine the use of Transcranial sonography in differentiating Parkinsonism syndromes. Park. Relat. Disord..

[10] Yan J.-H., Li K., Ge Y.-L., Li W., Wang P.-Z., Jin H., Zhang J.-R., Chen J., Wang F., Yang Y.-P. (2023). Quantitative Transcranial Sonography Evaluation of Substantia Nigra Hyperechogenicity Is Useful for Predicting Levodopa-Induced Dyskinesia in Parkinson Disease. Ultrasound Med. Biol..

[11] Garcia-Malo C., Vivian W., Carolina M., Peralta S.R., Lina A., Irene C.P., Juan J.G., Diego G.B. (2020). Quantitative transcranial sonography of the substantia nigra as a predictor of therapeutic response to intravenous iron therapy in restless legs syndrome. Sleep Med..

[12] Godau J., Sojer M. (2015). Transcranial sonography in restless legs syndrome. Int. Rev. Neurobiol..

[13] Garcia-Malo C., Novo-Ponte S., Castro-Villacanas Farzarnia A., Boi S., Castillo C.M., Peralta S.R., Vidal V.M., Botta L., Anguizola S., Cano-Pumarega I. (2021). Correlation between systemic iron parameters and substantia nigra iron stores in restless legs syndrome. Sleep Med..

[14] Wanner V., Garcia-Malo C., Miranda C., Krakowiak M.J., Cano-Pumarega I., Peralta S.R., Garcia-Borreguero D. (2019). Transcreal sonography as a novel neuroimaging tool to determine brain iron deficiency in restless legs syndrome: Results in a chilean sample. Sleep Med..

[15] Ruth T., Jennifer M., Michel R., Sophia G., James O., Thomas F., Maciej J.Z., Mary M., Leslie A.O., Nneka N. (2025). Pharmaceutical Practice Considerations Regarding Adoption of the Race-Free Chronic Kidney Disease Epidemiology Collaboration (CKD-EPI) 2021 Equations. Am. J. Kidney Dis..

[16] Allen R.P., Picchietti D.L., Garcia-Borreguero D., Ondo W.G., Walters A.S., Winkelman J.W., Zucconi M., Ferri R., Trenkwalder C., Lee H.B. (2014). Restless-legs syndrome/Willis–Ekbom disease diagnostic criteria: Updated International Restless Legs Syndrome Study Group (IRLSSG) consensus criteria—History, rationale, description, and significance. Sleep Med..

[17] Walter U., Skoloudík D. (2014). Transcranial sonography (TCS) of brain parenchyma in movement disorders: Quality standards, diagnostic applications and novel technologies. Ultraschall Med.—Eur. J. Ultrasound.

[18] Li X., Allen R.P., Earley C.J., Liu H., Cruz T.E., Edden R.A., Barker P.B., van Zijl P.C. (2016). Brain iron deficiency in idiopathic restless legs syndrome measured by quantitative magnetic susceptibility at 7 tesla. Sleep Med..

[19] Kalantar-Zadeh K., Block G., McAllister C.J., Humphreys M.H., Kopple J.D. (2004). Appetite and inflammation, nutrition, anemia, and clinical outcome in hemodialysis patients. Am. J. Clin. Nutr..

[20] Fishbane S., Paganini E.P. (2004). Iron management in hemodialysis patients. Semin. Dial..

[21] Avni T., Reich S., Lev N., Gafter-Gvili A. (2019). Iron supplementation for restless legs syndrome–A systematic review and meta-analysis. Eur. J. Intern. Med..

[22] Godau J., Wevers A.-K., Gaenslen A., Di Santo A., Liepelt I., Gasser T., Berg D. (2008). Sonographic abnormalities of brainstem structures in restless legs syndrome. Sleep Med..

[23] Richter D., Woitalla D., Muhlack S., Gold R., Tönges L., Krogias C. (2018). Brainstem raphe alterations in TCS: A biomarker for depression and apathy in Parkinson’s disease patients. Front. Neurol..

[24] Mislav B., Dalibor K., Zlatko T., Arijana L.H., Vlasta V., Jelena B., Vida D. (2010). Brainstem raphe lesion in patients with major depressive disorder and in patients with suicidal ideation recorded on transcranial sonography. Eur. Arch. Psychiatry Clin. Neurosci..

[25] Becker G., Berg D., Lesch K.P., Becker T. (2001). Basal limbic system alteration in major depression: A hypothesis supported by transcranial sonography and MRI findings. Int. J. Neuropsychopharmacol..

[26] Skoloudík D., Walter U. (2010). Method and validity of transcranial sonography in movement disorders. Int. Rev. Neurobiol..

